# Effective oral function improvement by restoration-driven implant treatment after mandibular resection with a scapular flap: a case report

**DOI:** 10.1186/s40729-022-00461-z

**Published:** 2022-12-07

**Authors:** Takayuki Kosaka, Masahiro Wada, Suzuna Akema, Yuichi Nishimura, Kazuhide Matsunaga, Narikazu Uzawa, Kazunori Ikebe

**Affiliations:** 1 Department of Prosthodontics, Gerodontology and Oral Rehabilitation, Osaka University Graduate School of Dentistry, 1-8 Yamadaoka, Suita, Osaka 565-0871 Japan; 2 Department of Oral and Maxillofacial Surgery II, Osaka University Graduate School of Dentistry, 1-8 Yamadaoka, Suita, Osaka 565-0871 Japan

**Keywords:** Mandibulectomy, Scapular flap, Implant, Mastication, Quality of life

## Abstract

**Background:**

The extensive loss of teeth and surrounding tissues due to mandibulectomy for an oral tumor not only impacts negatively on appearance, but also often causes various functional disorders, decreasing quality of life (QOL). In the present case, reconstruction with a scapular flap was carried out along with segmental mandibulectomy, aiming for functional restoration through restoration-driven implant treatment. A good outcome was obtained, with improvement of masticatory function and QOL following the prosthetic treatment.

**Case presentation:**

The patient was a 37-year-old woman diagnosed with ossifying fibroma in the left side of the mandible. Segmental mandibulectomy and reconstruction with a scapular flap were carried out. Implant diagnostic simulation was performed, and based on the result, secondary reconstruction using a particulate cancellous bone and marrow graft was carried out by an oral surgeon. After wound healing was complete, implant placement was performed twice, and the final prosthodontic treatment was completed. Masticatory performance and maximum bite force, which are indices of masticatory function, were improved from before to after prosthetic treatment. In addition, oral health-related QOL was improved from before to after prosthetic treatment.

**Conclusion:**

In the present case, restoration-driven implant treatment was performed in a patient following segmental mandibulectomy for a mandibular tumor, with a good outcome. Planning the treatment measures with a focus on the final prosthetic vision can lead to improvement of oral function in patients with extensive mandibular defects.

## Background

In cases of mandibulectomy for an oral tumor, the extensive loss of teeth and surrounding tissues not only impacts negatively on appearance, but also often causes various functional disorders of mastication, swallowing, and articulation [1], decreasing quality of life (QOL) [2]. Reconstruction of mandibular bone defects using autogenous bone is an effective means to improve not only the lost mandible morphology, but also oral function, and thus improve the patient’s QOL.

The first choice for reconstruction in cases of mandibulectomy is often a vascularized free flap using bone from the ilium, fibula, scapula, etc. [3]. Of these, the scapular flap offers extremely good blood circulation in both the cutaneous flap and the osseous flap, and the richly branched vascular pattern allows a high degree of flexibility in its use, making it suitable for almost all types of mandibular reconstruction, except extremely extensive defects [4].

However, full recovery of appearance and oral function after mandibulectomy can only be achieved with appropriate postoperative prosthetic treatment. An implant prosthesis can give a high level of patient satisfaction in terms of both esthetics and function [5, 6], but it requires a sufficient quantity of bone, and adequate simulation is essential before proceeding with treatment. Thus, restoration-driven implant treatment, in which the form of the final prosthetic device is decided and the form is achieved through secondary reconstruction of the bone followed by an implant prosthesis, is effective for ensuring the success of an implant prosthesis for mandibular defect. At the same time, it is important to perform enough evaluation of the oral function and patient satisfaction before and after surgery.

In the present case, a case of segmental mandibulectomy was treated by carrying out reconstruction with a scapular flap, aiming for functional restoration through restoration-driven implant treatment. A good outcome was obtained, with improvement of masticatory function and QOL following the prosthetic treatment.

## Case presentation

**Fig. 1:**
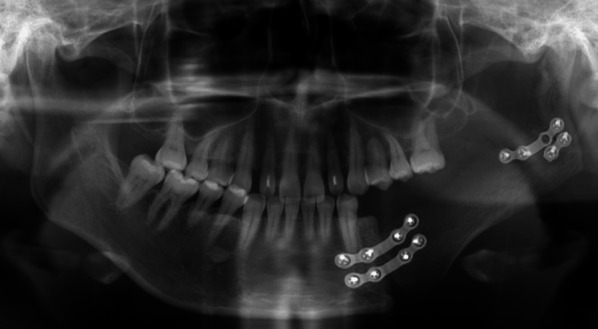
Panoramic radiograph after segmental mandibulectomy and reconstruction

The patient was a 37-year-old woman. She was diagnosed with ossifying fibroma in the left side of the mandible, for which segmental mandibulectomy and scapular flap reconstruction were carried out in October 2018 at the Division of Oral and Maxillofacial Disease, Osaka University Dental Hospital (Fig. 1). First, an incision was made in the left submandibular region, and the left inferior margin of the mandible was identified. The left mandibular first premolar was then extracted, and segmental mandibulectomy of the region posteriorly from the left first premolar and including the muscular process and the mandibular angle was performed. The left scapular flaps were a cutaneous flap of 13 × 5 cm^2^ and an osseous flap of 8 × 2.5 cm^2^, which were lifted with vascularization. The cutaneous flap and the osseous flap were adjusted in size to match the extent of the defect and implanted into the bone defect. For fixation of the osseous flap to the mandible, 2 titanium 4-hole miniplates were used at the mandibular body (anterior) and 1 4-hole miniplate and 1 2-hole miniplate were used at the mandibular branch (posterior). For revascularization of the cutaneous flap, the circumflex scapular artery in the flap was anastomosed to the left facial artery on the neck side, and the circumflex scapular vein was anastomosed to the left facial vein on the neck side to restore blood flow. The defect in the oral cavity was closed with the scapular cutaneous flap.

**Fig. 2:**
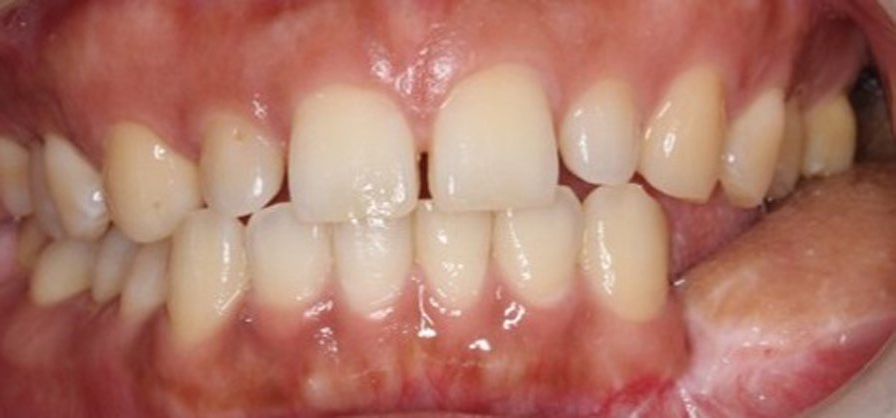
Intraoral view before prosthetic treatment

**Fig. 3:**
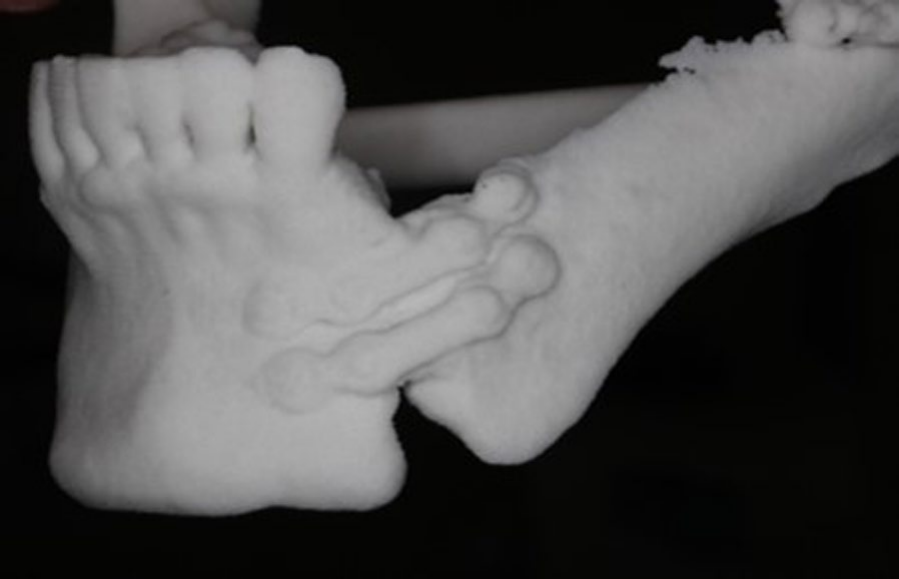
Three-dimensional plaster model of the mandible before secondary bone reconstruction

As a result of the surgery, the teeth from the mandibular left first premolar to the left second molar were missing, with all other teeth except the bilateral maxillary second molars remaining (Fig. 2). After healing of the wound, prosthetic treatment of the missing mandibular left molars started at the Division of Prosthodontics, Osaka University Dental Hospital. First, a three-dimensional plaster model of the mandible was made from multidetector-row computed tomography (MDCT) imaging data (Fig. 3), and the location and amount of bone growth in the secondary reconstruction were simulated in order to explore the best site for implant placement. Specifically, implant diagnostic simulation software (Landmark System, iCAT Corp., Osaka, Japan) was used to determine the ideal implant superstructure for occlusion on the basis of the position of opposing teeth, and virtual implants were placed directly under the superstructure. In the present case, the buccolingual width of the reconstructed area was significantly reduced as a result of the osseous flap reconstruction, and all of the virtual implants deviated from the bone. Given this situation, the quantity of bone ideally needed to support the implant was planned. The area corresponding to the lower left first premolar was close to the boundary between the mandibular bone and the grafted bone; to avoid implant placement at this site, a proximal cantilever bridge with implants at the left mandibular second premolar and first molar as abutments was planned for the final implant prosthetic device.

**Fig. 4:**
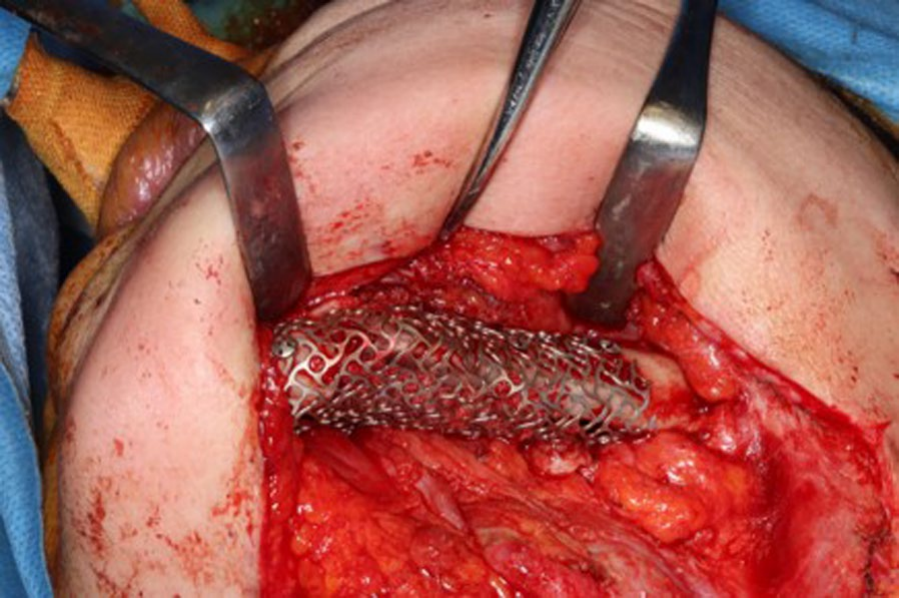
Secondary reconstruction using a PCBM graft covered by a titanium mesh plate

Secondary reconstruction using a particulate cancellous bone and marrow (PCBM) graft was performed by an oral surgeon in February 2020 on the basis of the results of the above simulation. First, an incision was made in the lower part of the left side of the mandible, and the inferior left margin of the mandible and the inferior margin of the scapula bone (the graft bone) were identified. The soft tissue attached to the graft bone was detached buccolingually to expose the whole area around the graft. Next, a titanium mesh plate that had been prepared in advance was placed, following the morphology of the bone from the inferior margin of the mandible and graft bone and fixed in position with screws (Fig. 4). In the bone cavity created between the titanium mesh plate thus fixed and the grafted bone on the lingual side, corresponding to the defect of the lingual alveolar bone from the left first premolar to the second molar, a sufficient amount of PCBM harvested from the left ilium was grafted to the height of the alveolar apex.

**Fig. 5:**
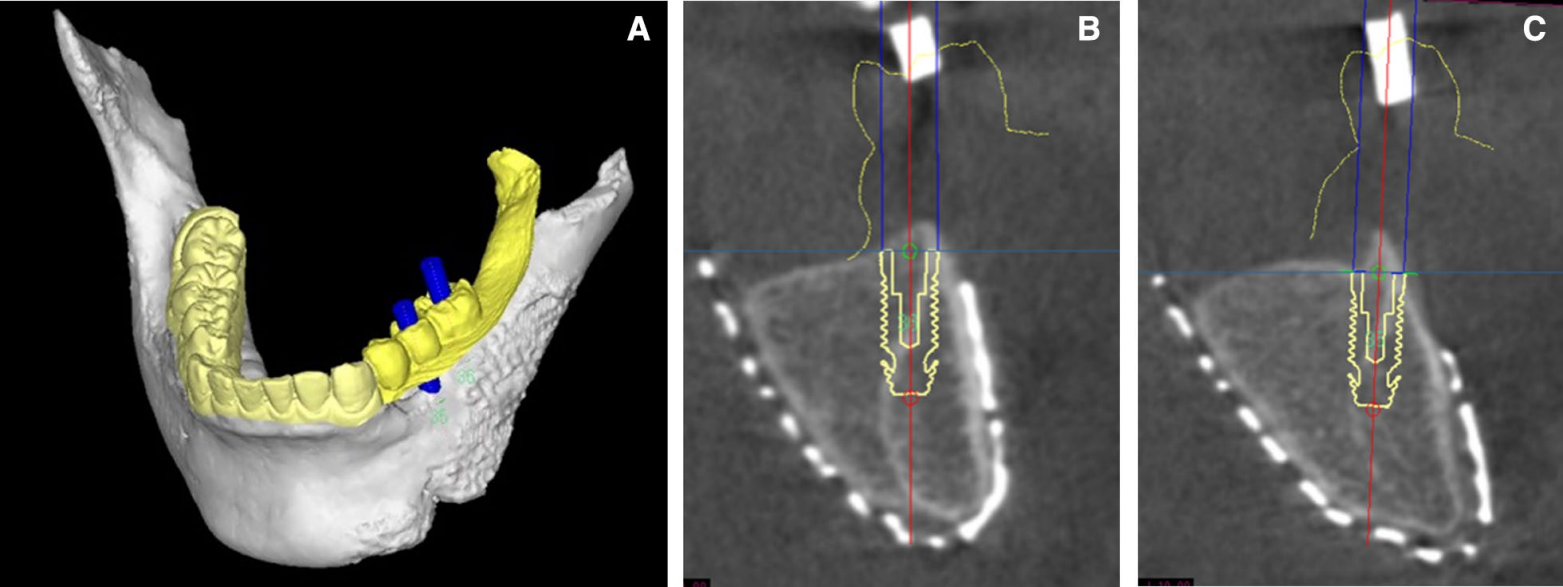
Final simulation of the implant placement using cone-beam CT image (**a**) and cross-sectional data of each implant position. Simulation of 36 implant (**b**) and 37 implant (**c**)

**Fig. 6:**
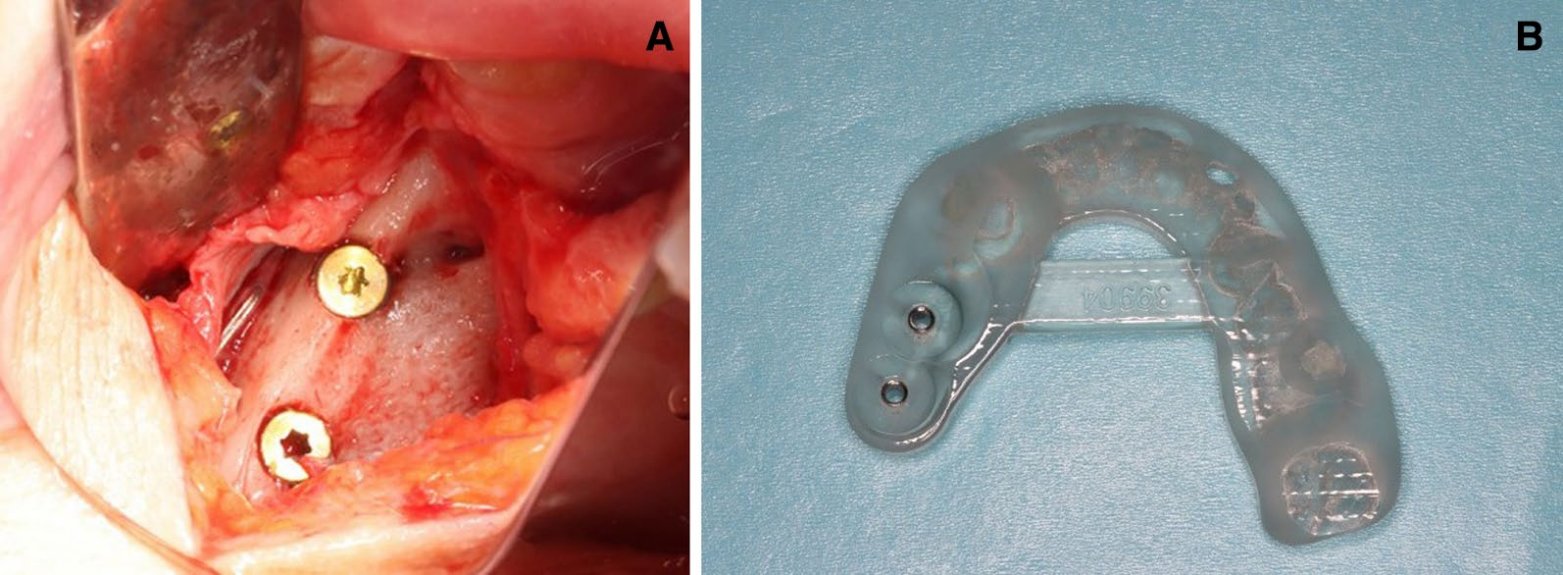
First implant placement surgery (**a**) with a surgical template (**b**)

After the wound had healed, cone-beam CT was carried out to reconfirm the quantity of grafted bone, and a final simulation of the implant placement was performed (Fig. 5). A surgical template was fabricated, and implant placement surgery was then performed. First, the oral surgeon opened the center of the cutaneous flap in the oral cavity to reveal the grafted bone, and the prosthetist then placed the implants (Genesio Plus; φ3.8*10 mm, GC Company, Tokyo, Japan) at the planned sites (35 and 36) (Fig. 6). Primary stability of each implant was under 20 Ncm because the bone density of the implant sites augmented by PCBM was relatively low.

**Fig. 7:**
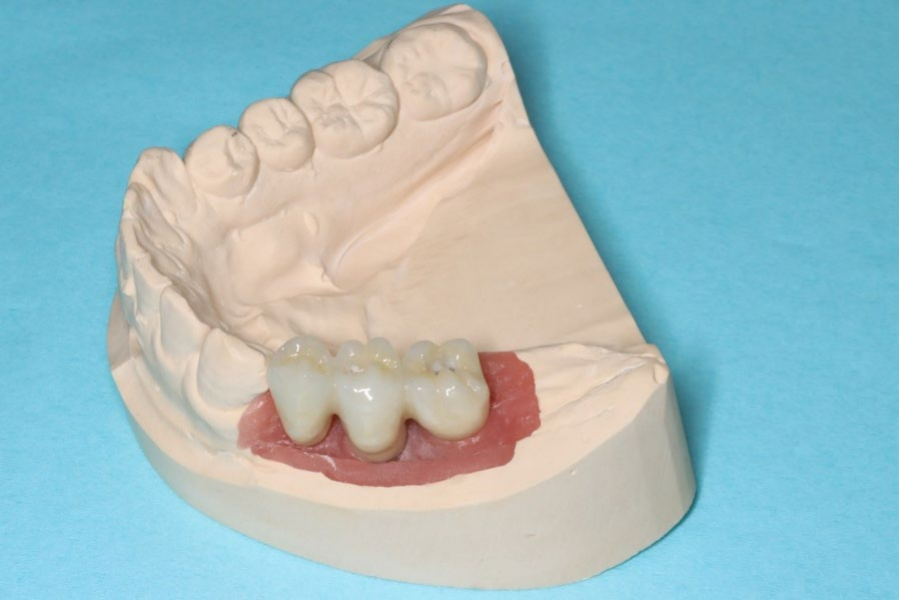
Fixed partial denture with a zirconia framework

**Fig. 8:**
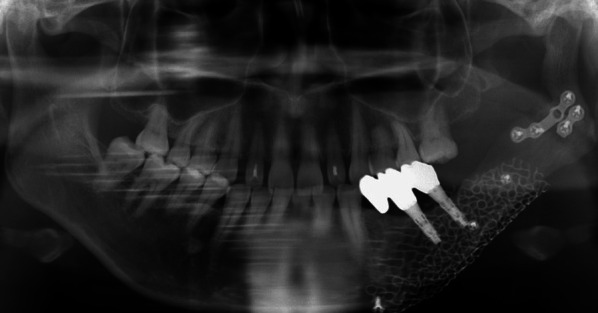
Panoramic radiograph after prosthetic treatment

**Fig. 9:**
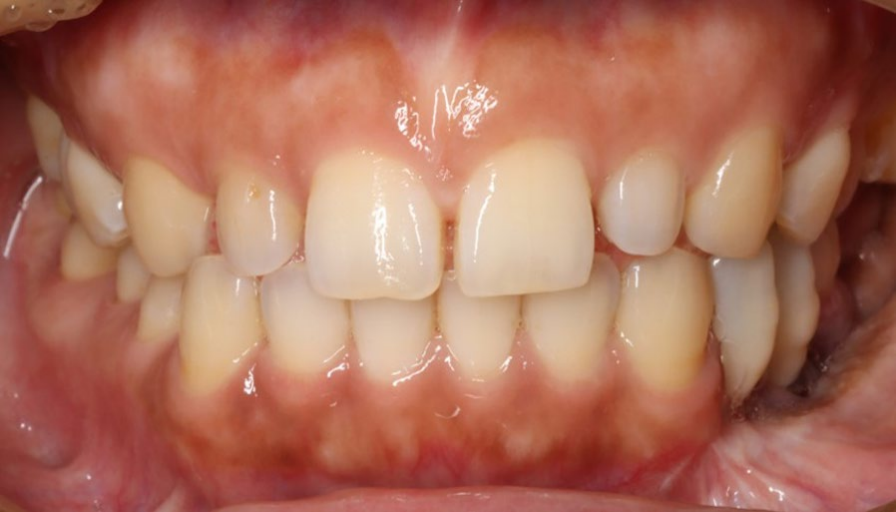
Intraoral view after prosthetic treatment

**Fig. 10:**
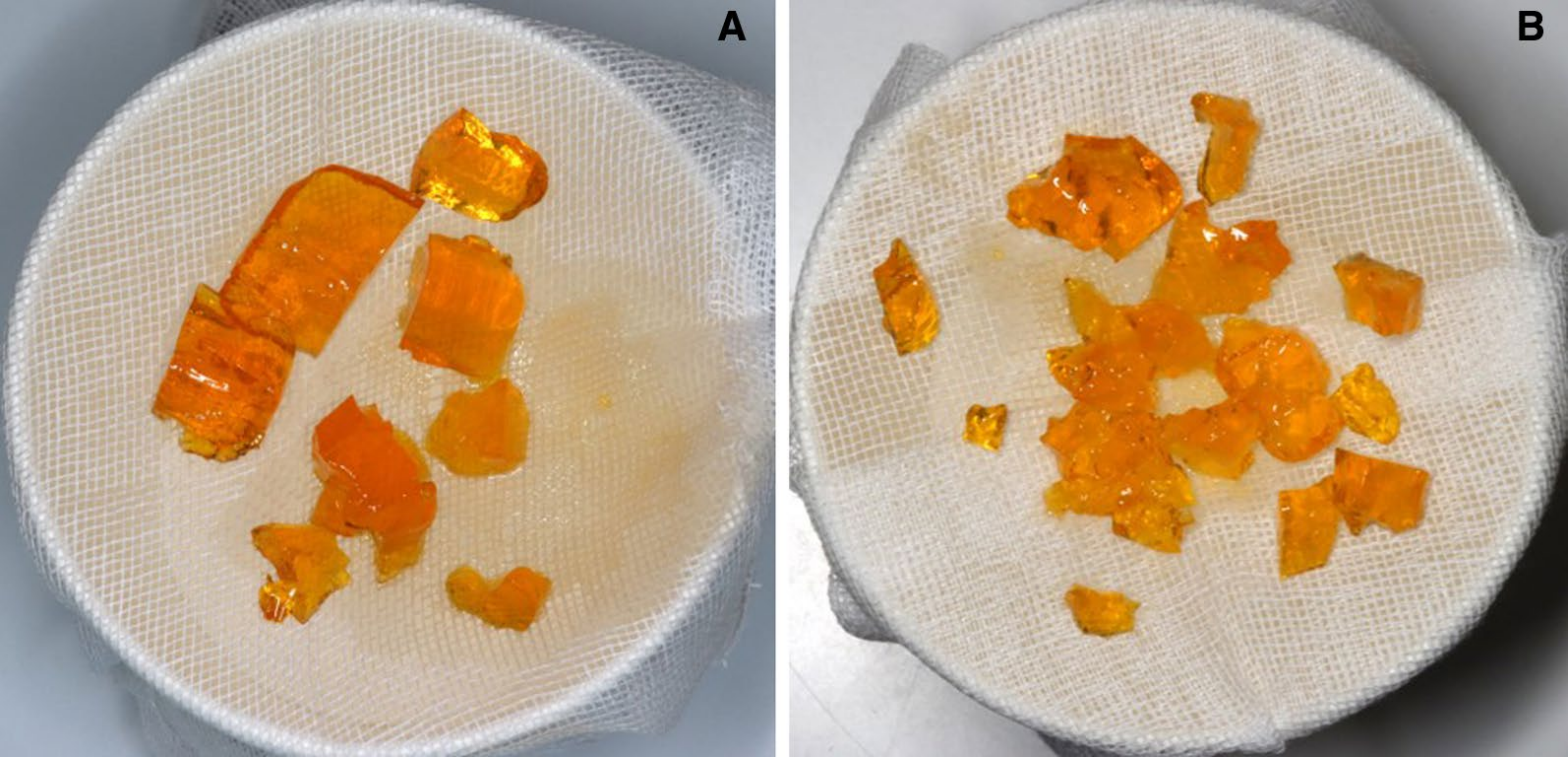
Masticatory performance examination before (**a**) and after (**b**) prosthetic treatment

**Fig. 11:**
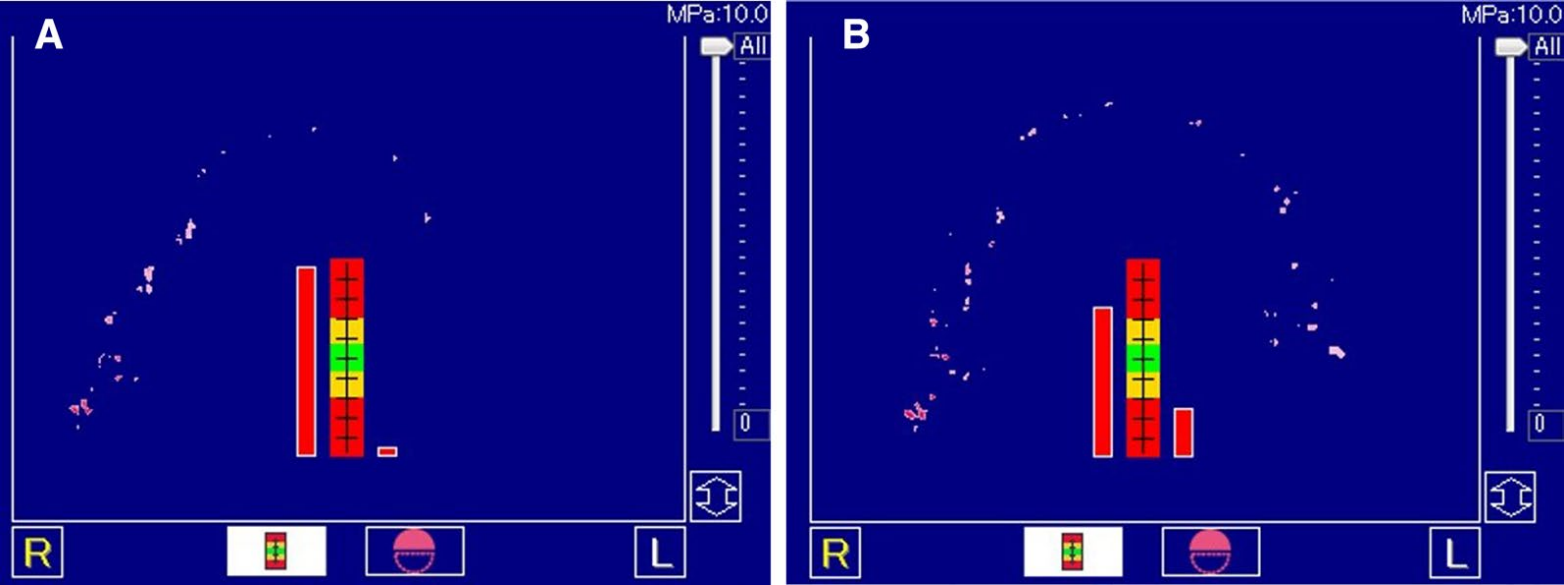
Maximum bite force examination before (**a**) and after (**b**) prosthetic treatment

After a 5-month unloading period, a second surgical procedure was performed to place the abutment and healing cap. Placement of the implant superstructure was judged to be difficult because the intraoral cutaneous flap had become thicker due to subcutaneous fatty tissue; therefore, the oral surgeon performed alveolar ridge plasty by reducing the amount of subcutaneous fatty tissue on the cutaneous flap and correcting the shape of the flap. The abutment and healing cap were then placed by the prosthetist. After waiting for the wound from the second procedure to heal, impressions were taken using the open-tray method, and provisional restorations were placed. After placement, peri-implant cleanliness and the occlusal relationship were carefully checked, precise impressions were taken again using the open-tray method, and a fixed partial denture fabricated by monolithic zirconia was tighten by fixing screws (Figs. 7, 8, 9). To evaluate masticatory function, masticatory performance (Soshaku-noryoku sokuteiyou gummy jelly, UHA Mikakuto, Osaka, Japan) and maximum bite force (Dental Prescale II, GC, Tokyo, Japan) were evaluated before and after prosthetic treatment. Masticatory performance improved from a pre-prosthetic treatment score of 4 to a post-treatment score of 6 (Fig. 10), and maximum bite force improved from a pre-treatment value of 398.8N to a post-treatment value of 658.4N (Fig. 11). In addition, oral health-related QOL was evaluated using the Oral Health Impact Profile-14 (OHIP-14), which improved from a pre-treatment score of 15 to a post-treatment score of 1.

## Discussion

In the present case, placement of a fixed prosthetic device by means of implants was selected for prosthetic treatment following extensive resection of the mandible due to an oral tumor. A simulation was performed to determine the optimal implant sites, and secondary reconstruction was carried out for the bone growth necessary for the implants. As a result, an appropriate occlusal relationship at the mandibular defect site was created.

To date, there have been numerous reports of cases of implant treatment for mandibular defects following mandibulectomy. In most of these, however, reconstruction using fibular bone and a plate was performed followed by implant therapy on the grafted bone, which is by no means sufficient for implants. In the present case, the desirable implant placement sites were considered in advance by envisaging the final appearance and occlusal status, after which sufficient bone volume was made up by secondary reconstruction. This allowed placement of the implant device at the optimal implant site. Guided bone regeneration (GBR), in which regeneration of bone tissue into areas of insufficient bone tissue is promoted, is routinely performed for implant treatment at many dental clinics [7]. However, in cases such as the present one with an extensive mandibular defect, and reconstruction is carried out with a cutaneous flap that has different properties from normal alveolar ridge mucosa, adjustment of the volume of the cutaneous flap and careful control of the volume of grafted bone are needed in the secondary reconstruction. An approach that combines specialists in oral surgery and specialists in prosthetics collaborating together needs to be put in place.

In the present case, a plan with the aim of restoring not only the patient’s appearance, but also her masticatory function, which were lost as a result of mandibulectomy, was drawn up. There are numerous methods available for evaluating a patient’s masticatory function, but it is important that the evaluation is objective and quantitative. In the present case, the method of evaluating the degree of comminution using gummy jelly intended for measurement of masticatory ability was used. In this method, the gummy jelly is freely chewed 30 times, and the degree of comminution is visually evaluated by comparing it against a score table. [8] Since this method allows anyone to easily evaluate masticatory performance, it is often used not just for clinical evaluations, but also in other settings, such as epidemiological research [9, 10]. In the present case, occlusion in the right molar region was established before the prosthetic treatment, so that a certain degree of masticatory function was retained. The patient herself stated that she somehow managed to eat by biting with the rear teeth on the right side. However, her masticatory performance improved from a pre-treatment score of 4 to a post-treatment score of 6, and she stated that, after the prosthetic treatment, she was able to chew food into small pieces and that meals took less time. Thus, it is essential to establish occlusion with a balance between both sides in the molar region to ensure smooth masticatory function, and this was achieved in the present case. Maximum bite force was also evaluated in the present case as an objective indicator of masticatory performance using the Dental Prescale system. In this method, a film for measuring occlusal force is placed between the upper and lower teeth, and the contact status of the teeth and the total pressure exerted by the teeth under maximum occlusal pressure at the maximal intercuspal position are evaluated [11]. It has been shown that maximum bite force correlates strongly with masticatory performance [12]. The establishment of occlusal support on the left side and the ability to exert occlusal pressure on the bilateral molars resulted in an improvement in maximum bite force, leading to improved masticatory performance.

When judging the results of dental treatment, it is important to evaluate the subjective level of satisfaction, as well as objective measures of oral function. In the present case, oral health-related QOL was evaluated as an indicator of the subjective level of satisfaction. The results showed that oral health-related QOL improved after the prosthetic treatment. Various other scales in addition to the OHIP-14 have been developed for evaluating oral health-related QOL, including the OHIP-49 [13] and the General Oral Health Assessment Index (GOHAI) [14], and these are widely used in clinical and research settings. The OHIP-14 is a short version of the OHIP-49, and it was used for the present case, because, in addition to being convenient to use, its reliability and validity have been reported [15]. Since improvement was seen following prosthetic treatment not just in the objective measure of masticatory function, but also in the subjective level of satisfaction, it is clear that the results of the prosthetic treatment in the present case were good.

In the present case, restoration-driven implant treatment was performed in a patient following segmental mandibulectomy for a mandibular tumor, with a good outcome. In addition, it was possible to visualize and accurately judge the effects of the treatment by conducting objective evaluation of masticatory function and subjective evaluation of the patient’s level of satisfaction before and after the prosthetic treatment. Planning the treatment measures with a focus on the final prosthetic vision while making use of the expertise of specialists from different fields, as in the present case, can lead to improvement of oral function in patients with extensive mandibular defects. At the same time, the soft tissue around implant was skin graft, which is completely different with normal oral mucosa. In this case, we did not perform mucosal transplantation such as free gingival graft or vestibuloplasty. Therefore, strict maintenance protocol including professional care and oral hygiene instruction is applied to this patient. It thinks to be better to obtain the keratinized tissue around implant to ensure better cleanability around implant prosthesis if applicable.

## Data Availability

Not applicable.

## Abbreviations

QOL: Quality of life
MDCT: Multidetector-row computed tomography
PCBM: Particulate cancellous bone and marrow
OHIP: Oral health impact profile
GBR: Guided bone regeneration
